# Synthesis, Spectroscopic Investigations (X-ray, NMR and TD-DFT), Antimicrobial Activity and Molecular Docking of 2,6-Bis(hydroxy(phenyl)methyl)cyclohexanone

**DOI:** 10.3390/molecules200713240

**Published:** 2015-07-21

**Authors:** Assem Barakat, Hazem A. Ghabbour, Abdullah Mohammed Al-Majid, Saied M. Soliman, M. Ali, Yahia Nasser Mabkhot, Mohammed Rafi Shaik, Hoong-Kun Fun

**Affiliations:** 1 Department of Chemistry, College of Science, King Saud University, P. O. Box 2455, Riyadh-11451, Saudi Arabia; E-Mails: amajid@ksu.edu.sa (A.M.A.-M.); mohamedali.eg25@gmail.com (M.A.); yahia@ksu.edu.sa (Y.N.M.); mshaik@ksu.edu.sa (M.R.S.); 2 Department of Pharmaceutical Chemistry, College of Pharmacy, King Saud University, P. O. Box 2457, Riyadh 11451, Saudi Arabia; E-Mails: ghabbourh@yahoo.com (H.A.G.); hfun.c@ksu.edu.sa (H.-K.F.); 3 Department of Chemistry, Rabigh College of Science and Art, King Abdulaziz University, P. O. Box 344, Rabigh 21911, Saudi Arabia; E-Mail: saied1soliman@yahoo.com; 4 Department of Chemistry, Faculty of Science, Alexandria University, P. O. Box 426, Ibrahimia, Alexandria 21321, Egypt; 5 X-ray Crystallography Unit, School of Physics, Universiti Sains Malaysia, Penang 11800, Malaysia

**Keywords:** Aldol product, cyclohexanone, X-ray, TGA, DFT, antimicrobial activity, molecular docking

## Abstract

The synthesis of 2,6-bis(hydroxy(phenyl)methyl)cyclohexanone **1** is described. The molecular structure of the title compound **1** was confirmed by NMR, FT-IR, MS, CHN microanalysis, and X-ray crystallography. The molecular structure was also investigated by a set of computational studies and found to be in good agreement with the experimental data obtained from the various spectrophotometric techniques. The antimicrobial activity and molecular docking of the synthesized compound was investigated.

## 1. Introduction

The Claisen-Schmidt reaction (cross-Aldol reaction) is a condensation reaction of aldehydes and carbonyl compounds leading to β-hydroxycarbonyl compounds and has been playing an important role in synthetic organic chemistry [1]. The bis(arylmethylidene)cycloalkanone moiety is a novel and versatile pharmacophore, as compounds bearing this structural unit possess a broad spectrum of biological activities such as cytotoxicity [2,3], cancer chemo-preventive [4], HIV-1 integrase inhibitory [5,6], cholesterol-lowering [7], antiangiogenic [8], quinine reductase inducing [9], and anti-oxidant [10] properties. The bis(arylmethylidene)cycloalkanones are used as a precursor for synthesis of a new class of tricyclic thiazolo[3,2-*a*]thiapyrano[4,3-*d*]pyrimidines, spiropyrrolidines which are antimicrobial and antifungal agents [11], and related analogues as potential anti-inflammatory agents [12], and other bioactive and novel heterocycles [13,14]. Thus, the synthesis of bis(arylmethylidene)cycloalkanones has attracted the attention of synthetic organic/medicinal chemists. The general approach involves cross-Aldol condensation of a cycloalkanone with an aromatic aldehyde [15], commonly catalyzed by a base [16] or an acid [17] and other compounds [18,19]. The condensation can be carried out using versatile reagents, such as Cp_2_TiPh_2_ [20], Cp_2_ZrH_2_ [21], RuCl_3_ [22], bis(*p*-methoxyphenyl)telluroxide (BMPTO) [23], TiCl_3_(CF_3_SO_3_) [24], SmI_3_ [25,26], La_3+_-immobilized organic solid [27], Mg(HSO_4_)_2_ [28], KF-Al_2_O_3_ [29], BF_3_·OEt_2_ [30], FeCl_3_ [31], TMSCl/NaI [32], InCl_3_ [33], SOCl_2_ [34], TMSCl/Pd-C [35], K_2_CO_3_/PEG-400 [36], Yb(OTf)_3_ [37], Cu(OTf)_2_ [38], molecular I_2_ [39], Et_3_N in the presence of LiClO_4_ [40], silica chloride [41], 1-methyl-3(2-(sulfooxy)ethyl)-1*H*-imidazol-3-ium chloride [42] and silica-supported silicaphosphinoxide (silphox, [POCl_3-n_(SiO_2_)_n_]) or phosphorus pentoxide (P_2_O_5_/SiO_2_) as heterogeneous reagents [43]. Despite the improvements offered by these routes, most of the reactions suffer from reverse and/or side reactions resulting in low yields of the desired products. One can assume that one challenge with a conventional Aldol approach is the reversibility of the Aldol reaction due to the unstabilized Aldol product. One group solved this problem [44] by using *N*-bocamide where the boc moiety rearranges to stabilize the resulting hydroxyl anion forcing the reaction towards product formation. This approach though, is mostly limited to aromatic aldehydes and gives modest yields for aliphatic aldehydes.

In the view of the above mentioned facts and in continuation of our interest [45,46,47,48], the structure of 2,6-bis(hydroxy(phenyl)methyl)cyclohexanone **1** was unambiguously deduced by single-crystal X-ray diffraction technique and elemental analysis. Also, the DFT/B3LYP calculations have been performed to study the molecular structure characteristics of the studied compound. The electronic and spectroscopic properties of the studied compound have been predicted using the same level of theory. The TD-DFT calculations were used to predict the possible electronic transitions. Natural bond orbital (NBO) calculations were performed to predict the natural atomic charges and study the different intramolecular charge transfer (ICT) interactions occurring in the studied system. The NMR chemical shifts are calculated using the gauge including atomic orbital (GIAO) method and used to assign the experimental results. The bioactivity of the synthesized product was tested by anti-microbial activity assay and also the molecular docking was investigated.

## 2. Results and Discussion

### 2.1. Synthesis of Compound ***1***

**Scheme 1:**
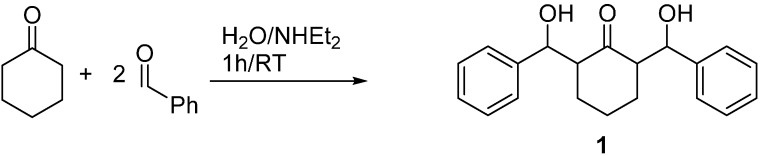
Preparation of the title compound **1**.

The title compound **1** was synthesized from a mixture of cyclohexanone and benzaldehyde (1:2) using aqueous diethylamine in excellent yield (85%) at room temperature for 1 h as depicted in Scheme 1.

The structure of compound **1** was confirmed by analysis of its spectroscopic data including FT-IR, ^1^H-, ^13^C-NMR and X-ray structure of single crystal.

### 2.2. Crystal Structure of Compound ***1***

**Figure 1:**
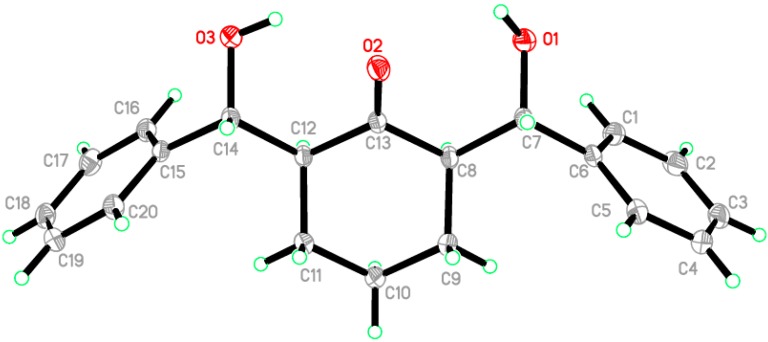
ORTEP diagram of the compound **1**. Displacement ellipsoids are plotted at the 50% probability level.

A clear colorless needle-like specimen of C_20_H_22_O_3_, approximate dimensions 0.68 × 0.15 × 0.14 mm, was used for the X-ray crystallographic analysis (Figure 1). The integration of the data using a monoclinic, *C*2/*c* unit cell yielded a total of 34,291 reflections to a maximum θ angle of 30.61, of which 4897 were independent (completeness = 99.8%, *R*_int_ = 3.1%, *R*_sig_ = 2.24%) and 3976 (81.19%) were greater than 2σ(*F*^2^). The final cell constants are *a* = 32.3262 (14) Å, *b* = 5.6987 (2) Å, *c* = 22.4859 (7) Å, β = 129.175 (2)°, volume = 3211.2 (2) Å^3^. Final refinement yields R[*F*^2^ > 2σ(*F*^2^)] = 0.048 and wR(*F*^2^) = 0.129 where w = 1/[σ^2^(*F_o_*^2^) + (0.0623*P*)^2^ + 3.520*P*] and *P* = (*F_o_*^2^ + 2*F_c_*^2^)/3. Data were corrected for absorption effects using the multi-scan method (SADABS). On the basis of the final model, the calculated density was 1.284 Mg/cm^3^ and *F*(*000*)1328 [49,50,51].

**Figure 2:**
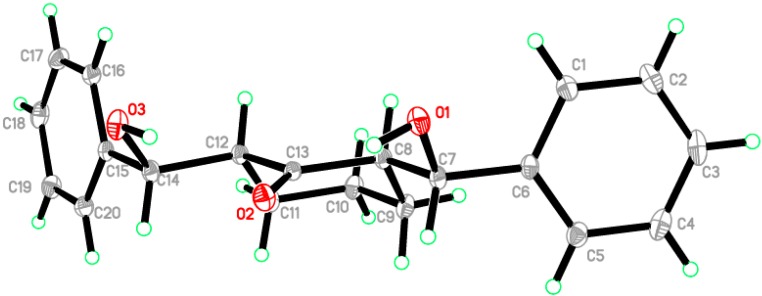
ORTEP diagram of the compound **1** drawn at 50%; ellipsoids showing the chair conformation of cyclohexanone ring.

**Figure 3:**
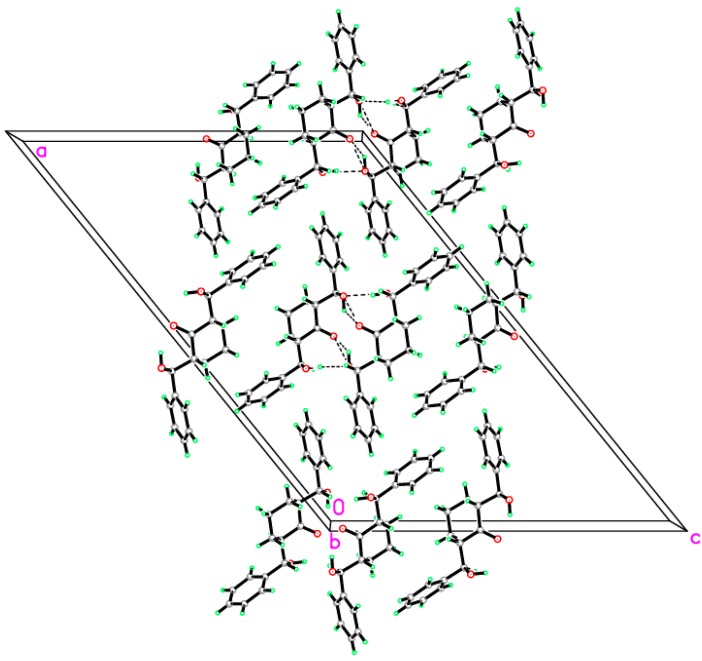
Crystal packing showing intermolecular C–H···O and N–H···O hydrogen bonds as dashed lines.

The asymmetric unit contains only one molecule of the compound. The molecular structure of compound **1** is composed of a cyclohexanone ring (C8–C13), which exhibits a chair conformation. The attached two hydroxyl methyl groups are disposed in equatorial configurations. (Figure 2). In the crystal structure, intermolecular O1—H1O1···O3 hydrogen bonds are observed (Figure 3 and Table 1 and Table 2). All geometric parameters (Å, °) and torsion angles of **1** are summarized in Tables S1 and 2 (Supplementary materials).

Table 1Crystal data of **1**.

**Table:** 

C_20_H_22_O_3_	*F*(000) = 1328
Mr = 310.37	*Dx* = 1.284 Mg m^−3^
Monoclinic, *C2/c*	*Z* = 8
*a* = 32.3262 (14) Å	Mo Kα radiation, λ = 0.71073 Å
*b* = 5.6987 (2) Å	Cell parameters from 9876 reflections
*c* = 22.4859 (7) Å	θ = 2.5–30.6°
β = 129.175 (2)°	µ = 0.09 mm^−1^
*V* = 3211.2 (2) Å^3^	T = 100 K
0.68 × 0.15 × 0.14 mm	Needle, colourless

**Table:** *Data collection*

Bruker APEX-II D8 Venture Diffractometer	3976 Reflections With I > 2σ(I)
Radiation source: Mo Kα radiation, λ = 0.71073 Å	*R*_int_ = 0.031
φ and ω scans	θ_max_ = 30.6°, θ_min_ = 2.3°
Absorption *correction*: multi-scan SADABS V2012/1 (Bruker AXS Inc., Billerica, MA,USA)	*h* = −45, 46
T_min_ = 0.91, T_max_ = 0.99	*k* = −7, 8
34291 measured reflections	*l =* −32, 31
4897 independent reflections

**Table:** *Refinement*

Refinement on *F*^2^	Hydrogen Site Location: Mixed
Least-squares matrix: full	H atoms treated by a mixture of independent and constrained refinement
R[*F*^2^ > 2σ(*F*^2^)] = 0.048	w = 1/[σ^2^(*F_o_*^2^) + (0.0623*P*)2 + 3.520*P*] where *P* = (*F_o_*^2^ + 2*F_c_*^2^)/3
wR(*F*^2^) = 0.129	(Δ/σ)max = 0.001
S = 1.03	Δρ_max_ = 0.62 e·Å^−3^
4897 reflections	Δρ_min_ = −0.19 e·Å^−3^
216 parameters	

**Table 2:** Hydrogen-bond geometry (Å, °) of **1**.

D—H···A	D—H	H···A	D···A	D—H···A
O1—H1O1···O3 ^i^	0.90 (2)	2.01 (2)	2.8799 (15)	162.1 (16)
O3—H1O3···O2	0.90 (3)	2.07 (2)	2.7835 (15)	136 (2)
O3—H1O3···O2 ^i^	0.90 (3)	2.27 (3)	2.8491 (17)	121.9 (18)

Symmetry code: (i) −x−1, −y, −z.

### 2.3. Computational Details

All the quantum chemical calculations of the studied compound were performed by applying DFT method with the B3LYP functional and 6‒311G(d,p) basis set using Gaussian 03 software [52]. The input file was taken from the CIF obtained from our reported X-ray single crystal measurement. The geometry was optimized by minimizing the energies with respect to all the geometrical parameters without imposing any molecular symmetry constraints. GaussView4.1 [53] and Chemcraft [54] programs have been used to draw the structure of the optimized geometry. The frequency calculations showed no negative values confirming that the optimized geometry is an energy minimum. The electronic spectra of the studied compound were calculated by the TD‒DFT method. The gauge including atomic orbital (GIAO) method was used for the NMR calculations. The ^1^H and the ^13^C isotropic shielding tensors referenced to the TMS calculations were carried out at the same level of theory. The Gaussian NBO 3.1 version were used to perform the natural bond orbital analyses [55].

#### 2.3.1. Optimized Molecular Geometry

**Figure 4:**
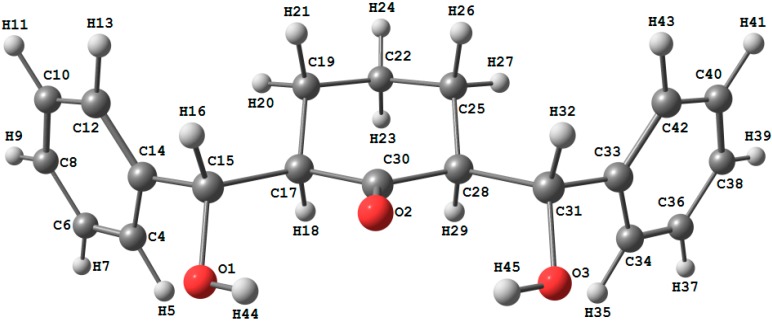

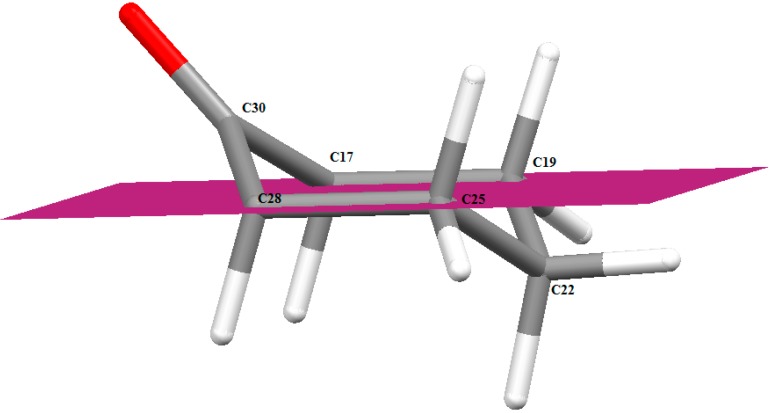
The optimized molecular structure of the studied compound (**upper**) and the chair form configuration of the cyclohexanone ring (**lower**).

**Figure 5:**
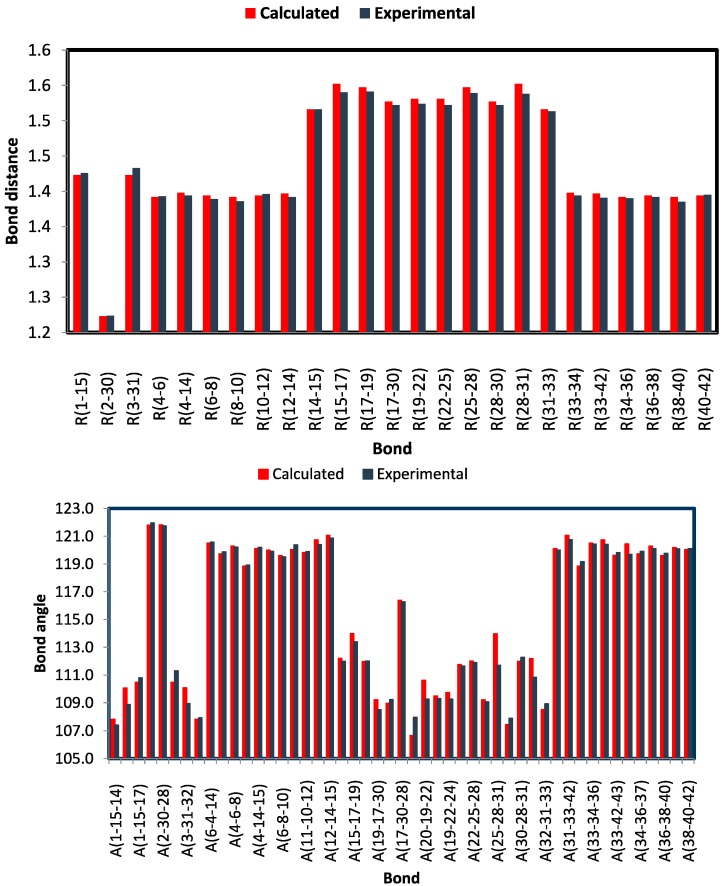
Comparison between the calculated and experimental geometric parameters (bond distances and angles) of the studied compounds.

The optimized bond lengths and bond angles obtained for the studied compound using the B3LYP method with 6‒311G(d,p) basis set are given in Table 3; while the atom numbering of the optimized structure is given in Figure 4. The studied compound possesses a C_1_ point group. The optimized geometry of the studied compound was compared with the structural parameters obtained from the crystallographic information file (CIF) as shown in Figure 5. Some of these geometric parameters are overestimated while others are underestimated. The deviations of the calculated bond length and bond angle values from the experimental data did not exceed 0.012–0.014 Å (C15‒Cl7 and C28‒C31) and 2.27° (C25‒C28‒C31) respectively. Generally, the bond lengths and bond angles are predicted very well. The calculated C‒C‒C bond angle values of the benzene rings are in the range of 118.8‒120.7° (exp. 118.9‒120.6°) [56]. The calculations predicted the O2...H45 intramolecular distance is 2.026 Å (exp. 2.068 Å) while the C30‒O2…H45 and O3‒H45…O2 bond angles are 99.6° (exp. 95.6°) and 131.2° (exp. 136.0°) respectively. These results indicate the presence of intramolecular O…H H‒bonding interactions. Moreover, the studied compound has three six membered rings, two are benzene and the third is cyclohexanone. The calculated C‒C‒C‒C dihedral angles of the two benzene rings do not exceed 1.0° indicating, commonly, the perfectly planar structure of these rings. In contrast, the cyclohexanone ring is not planar, whereas the C17, C19, C25 and C28 atoms are in the same plane. The C22 lies below this plane while the C30 above it; as a result the cyclohexanone ring has a chair configuration (Figure 4).

**Table 3:** The calculated and experimental geometric parameters of the studied compound using the DFT B3LYP/6-311G(d,p) method.

Parameter	Calc.	Exp	Parameter	Calc.	Exp
R(1-15)	1.423	1.426	A(7-6-8)	120.0	119.9
R(2-30)	1.223	1.224	A(6-8-10)	119.6	119.5
R(3-31)	1.423	1.433	A(8-10-12)	120.0	120.4
R(4-6)	1.392	1.393	A(11-10-12)	119.8	119.9
R(4-14)	1.398	1.394	A(10-12-14)	120.7	120.4
R(6-8)	1.394	1.389	A(12-14-15)	121.1	120.9
R(8-10)	1.392	1.386	A(14-15-17)	112.2	112.0
R(10-12)	1.394	1.396	A(15-17-19)	114.0	113.4
R(12-14)	1.397	1.392	A(15-17-30)	112.0	112.0
R(14-15)	1.516	1.516	A(19-17-30)	109.2	108.5
R(15-17)	1.552	1.540	A(17-19-21)	109.0	109.2
R(17-19)	1.547	1.541	A(17-30-28)	116.4	116.3
R(17-30)	1.527	1.522	A(20-19-21)	106.7	108.0
R(19-22)	1.531	1.524	A(20-19-22)	110.6	109.3
R(22-25)	1.531	1.522	A(19-22-23)	109.5	109.3
R(25-28)	1.547	1.539	A(19-22-24)	109.7	109.3
R(28-30)	1.527	1.522	A(19-22-25)	111.7	111.6
R(28-31)	1.552	1.538	A(22-25-28)	112.0	111.9
R(31-33)	1.516	1.513	A(25-28-30)	109.2	109.1
R(33-34)	1.398	1.394	A(25-28-31)	114.0	111.7
R(33-42)	1.397	1.391	A(29-28-31)	107.4	107.9
R(34-36)	1.392	1.390	A(30-28-31)	112.0	112.3
R(36-38)	1.394	1.392	A(28-31-33)	112.2	110.8
R(38-40)	1.392	1.385	A(32-31-33)	108.5	108.9
R(40-42)	1.394	1.395	A(31-33-34)	120.1	120.0
R(2-45)	2.026	2.068	A(31-33-42)	121.1	120.8
A(1-15-14)	107.8	107.4	A(34-33-42)	118.8	119.2
A(1-15-16)	110.1	108.9	A(33-34-36)	120.5	120.4
A(1-15-17)	110.5	110.8	A(33-42-40)	120.7	120.4
A(2-30-17)	121.8	122.0	A(33-42-43)	119.6	119.8
A(2-30-28)	121.8	121.7	A(35-34-36)	120.4	119.7
A(3-31-28)	110.5	111.3	A(34-36-37)	119.7	119.9
A(3-31-32)	110.1	108.9	A(34-36-38)	120.3	120.1
A(3-31-33)	107.8	107.9	A(36-38-40)	119.6	119.8
A(6-4-14)	120.5	120.6	A(39-38-40)	120.2	120.1
A(4-6-7)	119.7	119.9	A(38-40-42)	120.0	120.1
A(4-6-8)	120.3	120.2	A(30-2-45)	99.6	95.6
A(4-14-12)	118.8	118.9	A(3-45-2)	131.2	136.0
A(4-14-15)	120.1	120.2			

#### 2.3.2. Natural Atomic Charge

The calculated natural charges (NAC) at different atomic sites are given in Table 4. The O‒atoms are the most electronegative atomic sites in the molecule. The calculated natural charge densities at these atoms are in the range −0.6239 to −0.7486. The two oxygen atoms of the OH groups are more electronegative than the carbonyl oxygen. All the H-toms are electropositive, where the most electropositive H-sites are H44 and H45 (+0.4719) as these protons attached to the most electronegative O-atoms in the molecule. The rest of H-atoms have natural atomic charge values in the range of 0.1714–0.2258. Of these protons, the H18 and H29 are more electropositive than others. The H18 and H29 atoms are affected by the high electron withdrawing character of the C=O group. In contrast, the C-atoms have negative natural charges except C15, C30 and C31 which are bonded to O1, O2 and O3 atoms respectively. The carbonyl carbon (C30) has the highest positive NAC value.

**Table 4:** The natural atomic charges calculated at the B3LYP/6-311G(d,p).

Atom	NAC	Atom	NAC
O1	−0.7486	H24	0.2056
O2	−0.6239	C25	−0.3775
O3	−0.7486	H26	0.1879
C4	−0.1952	H27	0.2196
H5	0.2157	C28	−0.3173
C6	−0.1898	H29	0.2258
H7	0.2006	C30	0.6563
C8	−0.2010	C31	0.1467
H9	0.2003	H32	0.1714
C10	−0.1939	C33	−0.0406
H11	0.2005	C34	−0.1952
C12	−0.2057	H35	0.2157
H13	0.1993	C36	−0.1898
C14	−0.0406	H37	0.2006
C15	0.1467	C38	−0.2010
H16	0.1714	H39	0.2003
C17	−0.3172	C40	−0.1939
H18	0.2258	H41	0.2005
C19	−0.3776	C42	−0.2057
H20	0.2196	H43	0.1993
H21	0.1879	H44	0.4719
C22	−0.3674	H45	0.4719
H23	0.1897		

#### 2.3.3. Molecular Electrostatic Potential (MEP)

**Figure 6:**
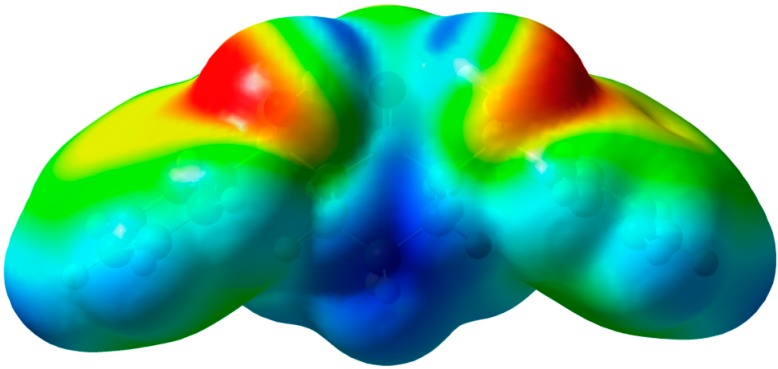
Molecular Electrostatic potentials (MEP) mapped on the electron density surface calculated by the DFT/B3LYP method.

Electrostatic potential maps are very useful three-dimensional diagrams used to visualize the charge distributions and charge related properties of molecules. The MEP is generated by overlapping the VdW radii of all atoms in the molecule so that its map reflects the molecule boundaries, and allows visualization of the size and shape of molecules. Also, the MEP picture has been used to predict the reactive sites for electrophilic and nucleophilic attack, and in studies of biological recognition and hydrogen bonding interactions [57,58].The MEP of the studied compound calculated using the B3LYP method with 6-311G(d,p) basis set is shown in Figure 6. The studied molecule has three O-atoms that might act as proton acceptor sites. It can be seen from this figure, that the negative regions (red) are mainly localized over the O-atoms of the hydroxyl groups. The O1 and O3 have more negative MEP values than the carbonyl oxygen (O2); hence O1 and O3 atoms are the most reactive towards the intermolecular H-bonding interactions as H-acceptor. In contrast, the maximum positive regions (blue) are distributed over the H-atoms (H44 and H45) which are the most reactive sites to nucleophilic attack [59]. From this point of view, the O1-H44 and O3-H45 are the most reactive H-donors for the intermolecular H-bonding interactions. In agreement with the reported X-ray structure the intermolecular O-H…O-H intermolecular H-bonding interactions are stronger than the OH···O=C one.

#### 2.3.4. Nonlinear Optical Properties

Nonlinear optical materials were used as key materials for photonic communications, which use light instead of electrons for data transmission. With the development of laser technology, nonlinear optical materials have been extensively applied to industry, national defense, medicine and research [60,61]. Several organic materials were used for such applications. These organic compounds were characterized by their high polarizability (α_0_) and low HOMO‒LUMO gap (ΔE). The α_0_ and ΔE values of the studied compound are calculated to be 220.17 Bohr^3^ and 5.3767 eV respectively. The polarizability of the studied compound is eight times higher than urea. In addition, the studied compound has a lower energy gap (ΔE) compared to urea. Based on these calculations, the studied molecule is considered as a better NLO material than this reference molecule used in literature [62].

#### 2.3.5. Frontier Molecular Orbitals

**Figure 7:**
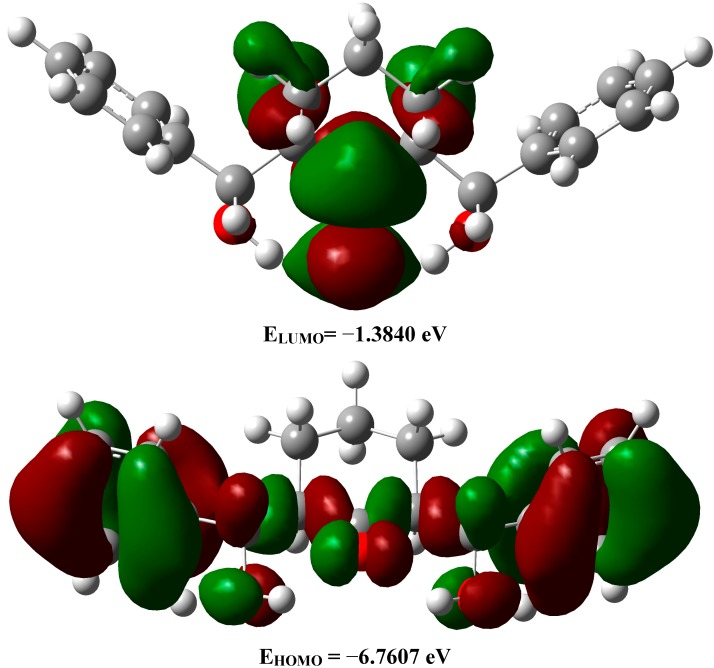
The ground state isodensity surface plots for the frontier molecular orbitals.

The properties of the frontier molecular orbitals (FMOs) are very useful for physicists and chemists. The electron densities of these FMOs were used for predicting the most reactive position in π‒electron systems and also explained several types of reactions in conjugated systems [63]. Moreover, eigenvalues of the lowest unoccupied molecular orbital (LUMO) and the highest occupied molecular orbital (HOMO) and their energy gap reflect the chemical reactivity of the molecule. Recently the energy gap between HOMO and LUMO has been used to prove the bioactivity from intramolecular charge transfer (ICT) [64,65]. The HOMO‒LUMO energy gap for the studied compound is calculated by B3LYP/6‒311G(d,p). The HOMO and LUMO pictures are shown in Figure 7. It is found that the HOMO level is mainly localized on the benzene ring π-system and extended to the C31-C28, C30=O2 and C17-C15 bonds. The LUMO is located mainly on the C=O group of the cyclohexanone ring. The E_HOMO_ and E_LUMO_ are calculated to be −6.7607 eV and −1.3840 eV respectively. The HOMO‒LUMO energy gap (ΔE) represents the lowest energy electronic transition. In the studied compound, the HOMO‒LUMO energy gap of the studied compound is 5.3767 eV (Figure 7). This electron transition belongs mainly to π‒π* excitation.

**Figure 8:**
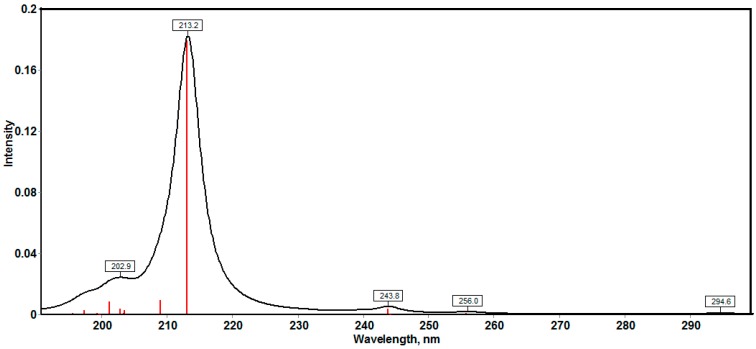
The calculated electronic spectra of the studied compound using the TD-DFT method.

The accurate electronic transitions were calculated using the time‒dependent density functional theory (TD‒DFT). The twenty spin allowed singlet-singlet electronic transitions calculated using the TD‒DFT method and are shown in Table S3 (Supplementary Information). The calculated electronic spectrum is shown in Figure 8. The calculations predicted electronic transitions bands at 202.9 nm, 213.2, 243.8 nm, 256 nm and 294.6 nm. The first two bands were observed experimentally at 204 and 231 nm, respectively. The latter is due to the electronic transitions from H→L+1 (71%) and H−4→L+1 (11%). The rest of the calculated electronic transition bands have low intensity and have no importance in the gas phase. In the experimental spectra, the longest wavelength transition band is observed at 328nm while calculated at 294.6 nm. The medium effects could be the main reason for the difference between the experimental and calculated values.

#### 2.3.6. NMR Spectra

**Figure 9:**
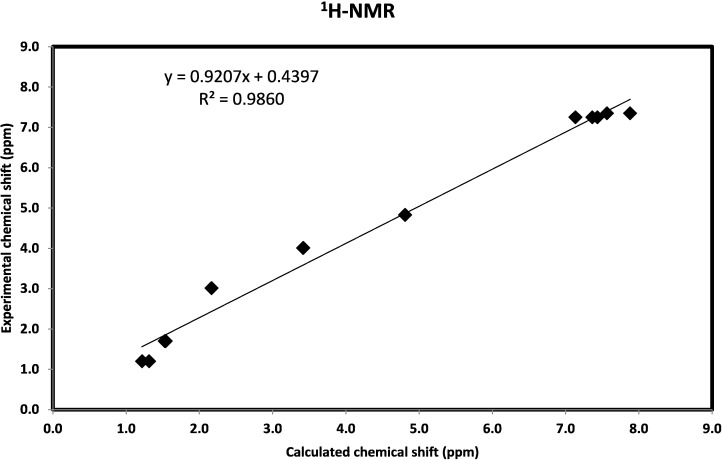

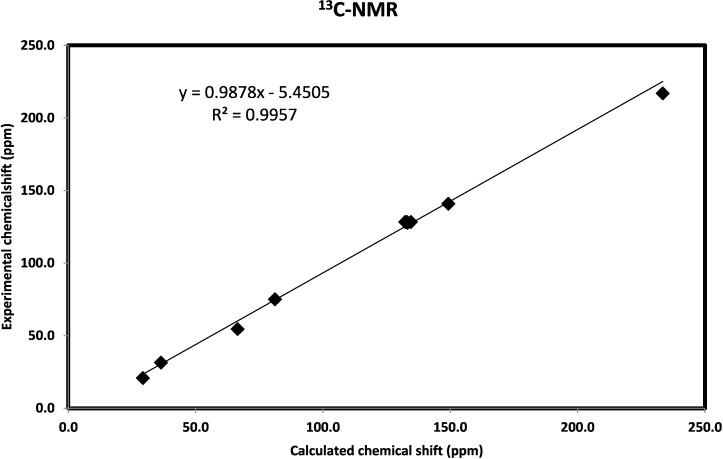
The correlation graph between calculated and experimental ^1^H-NMR and ^13^C-NMR chemical shifts of the studied compound.

The isotropic magnetic shielding (IMS) values calculated using the GIAO approach at the 6-311G(d,p) level are used to predict the ^13^C and ^1^H chemical shifts (δ_calc_) for the studied compound and the results are correlated to the experimental NMR data (δ_exp_) in CDCl_3_ solvent. The experimental and theoretical values for ^1^H- and ^13^C-NMR chemical shifts of the studied compound are given in Table S4 (Supplementary Information). According to these results, the calculated chemical shifts comply with the experimental findings. As shown in Figure 9, good correlations between the experimental and the calculated chemical shifts for carbon (R^2^ = 0.9957) and proton (R^2^ = 0.9860) were obtained.

#### 2.3.7. Natural Bond Orbital Analysis

The occupancy of electrons and p-character in significant natural bond orbitals (NBO) for the studied compound are given in Table S5 (Supplementary Information). It is noted that, the hydrogen atoms have almost 0% of p-character for all C–H and O–H bonds. In contrast, 100% *p*-character was observed in both the atoms in π-bonding of the C–C and C–O bonds. Similarly, almost 100% *p*-character was observed in the second lone pair of all O-atoms.

It is well known that the ideal sp^2^ hybrid orbital has 66.67% p-character while the ideal sp^3^ hybrid orbital has 75.00% p-character. The BD(1)C4-C6 orbital with 1.9770 electrons has 50.24% C4 character (64.06%p) and 49.76% C6 character (63.70%p). On the other hand, the BD(1)C17-C19 orbital with 1.9593 electrons has 51.39% C17 character (73.03%p) and 48.61% C19 character (72.38%p). The BD(1)O1-C15 orbital with 1.9891 electrons has 66.59% O1 character (69.79%p) and 33.41% C15 character (79.37%p). The BD(1)O2-C30 orbital with 1.9943 electrons has 67.29% O2 character (55.69%p) and 32.71% C30 character (71.04%p). The two C-atoms forming the C-C hybrids have almost the same % electron density (%ED). In contrast, the more electronegative O-atoms have higher electron density and lower %p characters than the C-atom bonded to it. The higher %ED on the O-atom is due to its higher electronegativity than carbon. As a result, the NBO analyses showed that all the C–O and C=O bond orbitals are polarized towards the oxygen atoms. The percentage polarization of the O-atoms for these bond hybrids are in the range of 81.60%–86.24%. Therefore, they provide higher electron density on the oxygen atoms, which is responsible for the polarity of the studied compound (3.9588D).

##### Second-Order Perturbation Theory

The natural bond orbital (NBO) calculations were performed in order to understand various interactions between the filled NBOs of one bond and vacant orbitals of another one, which is a measure of the intramolecular delocalization of electrons. The stabilization energies E^(2)^ deduced from the NBO calculations for the most significant intramolecular charge transfer interactions are reported in Table 5. The larger the E^(2)^ value, the more intensive is the interaction between electron donor and electron acceptor NBOs, *i.e*., the greater the extent of conjugation of the whole system [66]. The energy of these interactions can be estimated by the second‒order perturbation theory [67]. The ICT interactions formed by the orbital overlap between π→π* and n→σ* causing stabilization of the system up to 21.51 kcal/mol. The LP(2)O1→BD*(1)C15-C17, LP(2)O2→BD*(1)C17-C30, LP(2)O2→BD*(1)C28-C30 and LP(2)O3→BD*(1)C28-C31 ICT have the E^(2)^ values of 6.81, 18.04, 18.04 and 6.81 kcal/mol. These results indicate the presence of some electron delocalization from the second lone pair of each oxygen atom to the neighbouring C–C bonds. It seems that the ICT interactions from the LP(2)O2 of the carbonyl group are more intensive than that for the LP(2)O1 and LP(2)O3 of the hydroxyl groups. Moreover, the LP(2)O2→BD*(1)O1-H44/BD*(1)O3-H45 ICT interactions have stabilization energies of 2.69 and 2.70 kcal/mol respectively which indicate that the O···H intramolecular H-bonding are weak interactions.

**Table 5:** The second order perturbation energies E^(2)^ (kcal/mol) of the most important charge transfer interactions (donor–acceptor) of the studied compound using B3LYP method.

Donor NBO (i)	Acceptor NBO (j)	E^(2)^ kcal/mol
BD(2)C4-C6	BD*(2)C8-C10	20.76
BD(2)C4-C6	BD*(2)C12-C14	21.51
BD(2)C8-C10	BD*(2)C4-C6	20.08
BD(2)C8-C10	BD*(2)C12-C14	19.86
BD(2)C12-C14	BD*(2)C4-C6	19.53
BD(2)C12-C14	BD*(2)C8-C10	21.03
BD(2)C33-C42	BD*(2)C34-C36	19.53
BD(2)C33-C42	BD*(2)C38-C40	21.02
BD(2)C34-C36	BD*(2)C33-C42	21.51
BD(2)C34-C36	BD*(2)C38-C40	20.76
BD(2)C38-C40	BD*(2)C33-C42	19.86
BD(2)C38-C40	BD*(2)C34-C36	20.08
LP(2)O1	BD*(1)C15-C17	6.81
LP(2)O2	BD*(1)C17-C30	18.04
LP(2)O2	BD*(1)C28-C30	18.04
LP(2)O3	BD*(1)C28-C31	6.81
LP(2)O2	BD*(1)O1-H44	2.69
LP(2)O2	BD*(1)O3-H45	2.70

#### 2.3.8. Thermogravimetric Analysis (TGA)

**Figure 10:**
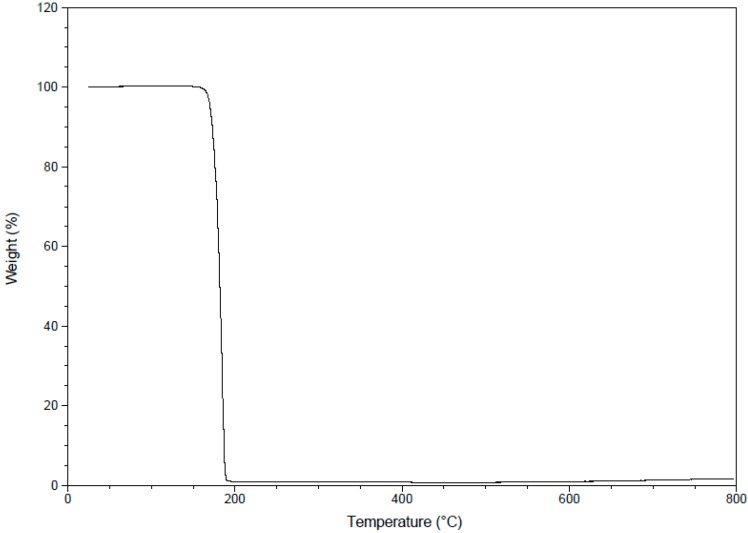
The TGA curve of the studied compound.

The TGA of the studied compound is performed over the temperature range 25–800 °C under a flowing nitrogen atmosphere and the result is shown in Figure 10. The TGA data showed that the studied compound is thermally stable up to 165 °C then undergoes sublimation without thermal decomposition in fast step leaving almost 0% residue.

### 2.4. Antimicrobial Evaluation

*In vitro* antimicrobial activity of the synthesized compound was investigated against microbial activity (two Gram-positive and two Gram-negative bacteria) and against fungal activity (four fungal species). Diameter of the inhibition zone was used as a criterion for the antimicrobial activity using a well diffusion agar method [68]. The standard antimicrobial agents were used to compare the potency of the tested compound. The results are summarized in Table 6 and Table 7.

**Figure 11:**
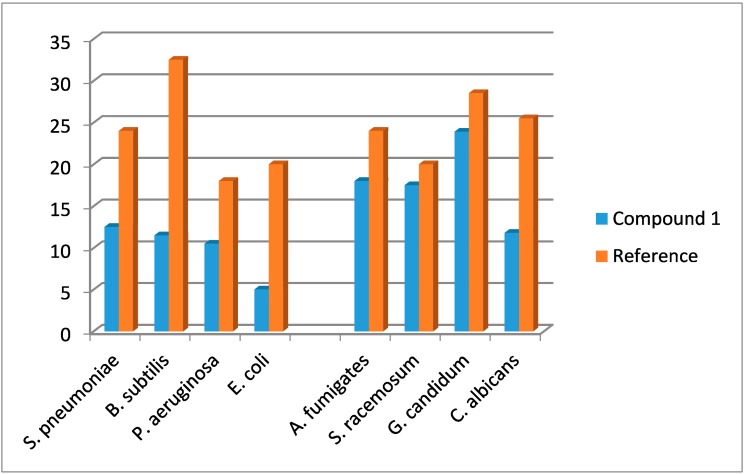
Inhibition zone results of the synthesized compound and the references drugs.

Results of the *in vitro* antibacterial activity revealed that compound **1** shows moderate activity against all tested bacteria (Table 6). Data from the antifungal evaluation have shown that compound **1** was also potent against the four tested fungal species (Table 7). Direct graph of inhibition zone results of the synthesized compound and the references drugs is shown in Figure 11.

**Table 6:** Antibacterial activity of the synthesized compound **1** (Zone of inhibition; diameter in mm).

Compound	Gram Positive Bacteria		Gram Negative Bacteria	
	*Streptococcus pneumoniae*	*Bacillissubtilis*	*Pseudomonas aeruginosa*	*Escherichia coli*
	Ampicillin		Gentamicin	
**Standard**	24 ± 0.45	32.5 ± 0.60	18.0 ± 0.15	20.0 ± 0.21
**1**	12.5 ± 0.31	11.5 ± 0.31	10.5 ± 0.41	5.0 ± 0.35

Data are expressed as mean ± SD; Standard = 25 μg/mL.

**Table 7:** Antifungal activity of the synthesized compound **1** (Zone of inhibition; diameter in mm).

Compound	Fungal Strains			
	*Aspergillus fumigates*	*Syncephalastrum racemosum*	*Geotricum candidum*	*Candida albicans*
	Amphotericin B			
**Standard**	24.0 ± 0.35	20.1 ± 0.15	28.5 ± 0.45	25.5 ± 0.35
**1**	18.0 ± 0.15	17.5 ± 0.40	23.9 ± 0.65	11.8 ± 0.45

Data are expressed as mean ± SD; Standard = 25 μg/mL.

### 2.5. Molecular Docking

**Figure 12:**
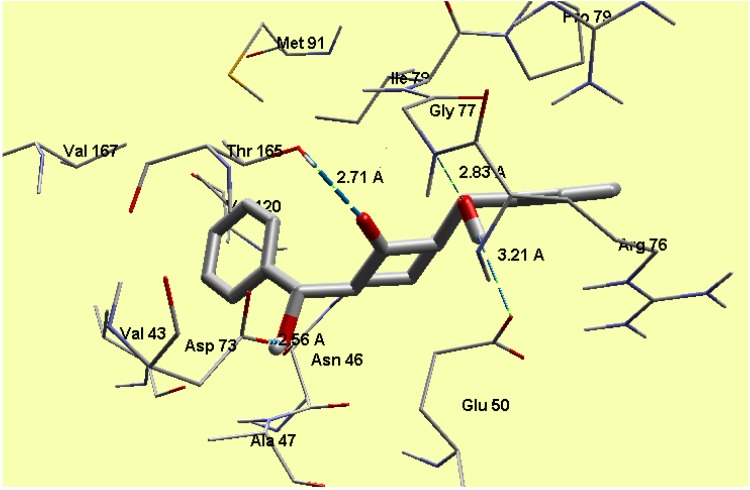
Interaction of compound **1** with the active site of *E. coli* 24 kda domain.

**Figure 13:**
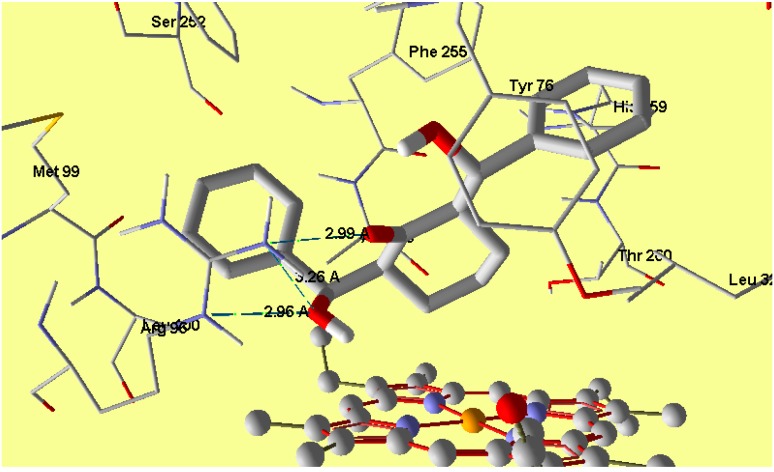
Interaction of compound **1** with the active site of cytochrome P450 14α-sterol demethylase.

In *E. coli*, the 24kda domain clorobiocin (reference compound) was found to have hydrogen bonding interactions with Asp73 (1.911 Å), Thr165 (2.109 Å), Asn46 (2.034 Å) and Arg136 (2.071 Å) with MolDock score −175.0. The compound **1** revealed a MolDock score −133.5 and formed four hydrogen bonding interactions with Thr165 (2.71 Å), Asp73 (2.56 Å), Gly77 (2.83 Å) and Glu50 (3.21 Å) (Figure 12) and this shows good interaction between the tested compound and the active site of the DNA gyrase enzyme, and also reveals a good docking result due to matching between the amino acids which interact with the tested and reference compound. Regarding the cytochrome P450 14𝛼-sterol demethylase, the compound **1** revealed a MolDock score −141.2 and formed three hydrogen bonding interactions with Arg96 (2.96, 2.99 and 3.26 Å) (Figure 13), which supports the biological results as seen by the good antifungal activity. The compound 1 is perpendicular to the porphyrin plane heme ion with nearest distance of 2.94 Å.

## 3. Experimental Section

### 3.1. General

All the chemicals were reagent-grade, purchased from Sigma-Aldrich and Fluka, among others, and were used without further purification, unless otherwise stated. All melting points were measured on a Gallenkamp melting point apparatus in open glass capillaries and are uncorrected. IR Spectra were measured as KBr pellets on a Nicolet 6700 FT-IR spectrophotometer. The NMR spectra were recorded on a Jeol-400 NMR spectrometer. ^1^H-NMR (400 MHz), and ^13^C-NMR (100 MHz) were run in deuterated chloroform (CDCl_3_). Chemical shifts (δ) are referred in terms of *ppm* and *J* -coupling constants are given in *Hz*. Mass spectra were recorded on a Jeol JMS-600 H. Elemental analysis was carried out on Elmer 2400 Elemental Analyzer in CHN mode. The X-ray diffraction measurement of compound **1** was collected by using Bruker SMART APEXII D8 Venture diffractometer. The thermal analysis of the studied compound has been carried out using TGA Q500 V20.10. The wt % loss has been measured from the ambient temperature up to 800 °C. The electronic spectrum of the studied compound is measured using Perkin Elmer, Lambda 35, UV/Vis spectrophotometer. The experimental electronic spectra is given in Supplementary data (Figure S4).

### 3.2. Preparation of 2,6-Bis(hydroxy(phenyl)methyl)cyclohexanone *(**1**)*

A mixture of cyclohexanone (1.5 mmol, 147 mg), benzaldehyde (3 mmol, 320 mg) and Et_2_NH (1.5 mmol, 155.5 μL) in 3 mL of degassed H_2_O was stirred at room temperature for 1 h until TLC showed complete disappearance of the reactants. 10 mL (1 M HCl) were added, extract with CHCl_3_, wash with brine, dried over MgSO_4_, evaporated and recrystallized from DCM/Et_2_O to afford the title compound **1**, as a colorless crystals (85%) , m.p. 110 °C; IR (ν_max_)(KBr)/cm^−1^ 3530, 3472, 3033, 2911, 2855, 1685, 1493, 1450, 1253, 1019; ^1^H-NMR (400 MHz; CDCl_3_) 7.25–7.35 (m, 10H, Ph), 4.83 (d, 2H, *J =* 8.6 Hz, CHOH), 4.01(brs, 2H, OH), 3.01 (m, 2H, CH), 1.2–1.7 (m, 6H, CH_2_); ^13^C-NMR (100 MHz; CDCl_3_) δ 217.06, 141.01, 128.26, 127.81, 75.06, 54.51, 31.36, 20.75.; MS *m*/*z* (%): 310.16 [M^+^, 98%]; Anal. calcd. for C_20_H_22_O_3_: C, 77.39; H, 7.14; Found: C, 77.41; H, 7.15. Uv-Vis (Ethanol): 204, 231 and 328 nm.

### 3.3. Agar Diffusion Well Method to Determine the Antimicrobial Activity

#### 3.3.1. Antifungal Activity of Compound **1**

Tested sample was screened *in vitro* for its antifungal activity against various fungi, namely, *Aspergillus fumigatus* (RCMB 002568), *Syncephalastrumracemosum* (RCMB 016001) *Geotricumcandidum* (RCMB 05097) and *Candida albicans* (RCMB 05036). The antifungal activity was performed by agar well diffusion method [68].

Fungal strains were grown in 5 mL Sabouraud dextrose broth (glucose/peptone; 40/10) for 3–4 days to obtain 10^5^ CFU/mL cells. The fungal culture (0.1 mL) was spread uniformly on the Sabouraud dextrose agar plates by sterilized triangular folded glass rod. Plates were left for 5–10 min so the culture was properly adsorbed on the surface. Then small wells 4 mm × 2 mm were cut into the plates with the help of well cutter and the bottoms of the wells were sealed with 0.8% soft agar to prevent the flow of test sample at the bottom of the well. 100 μL of the tested sample (10 mg/mL) was loaded into the wells of the plates. Compound **1** dissolved in DMSO, while pure DMSO was also used as control. The plates were kept for incubation at 30 °C for 3–4 days and then examined for the formation of zones of inhibition. The test was performed three times for each fungus and the average was taken. Amphotericin B was used as standard antifungal drug.

#### 3.3.2. Antibacterial Activity of Compound **1**

Antibacterial activities were investigated by using agar well diffusion method, against the *Staphylococcus pneumonia* (RCMB 010010) and *Bacillus subtilis* (RCMB 010067) (as Gram-positive bacteria) and *Pseudomonas aeruginosa* (RCMB 010043) and *Escherichia coli* (RCMB 0100052) (as Gram-negative bacteria). The solution of 10 mg/mL of compound in DMSO was prepared for testing against bacteria. Centrifuged pellets of bacteria from 24 h old culture containing approximately 10^4^–10^6^ CFU (colony forming unit) per mL were spread on the surface of nutrient agar (type tone 1%, yeast extract 0.5%, NaCl 0.5%, agar, and 1000 mL of distilled water, pH 7.0) which was autoclaved under 121 °C for at least 20 min. Wells were created in medium with the help of sterile metallic bores and then cooled down to 45 °C. The activity was determined by measuring the diameter of the inhibition zone (in mm). A volume of 100 μL of the tested sample (10 mg/mL) was loaded into the wells of the plates. A solution of the compound was prepared in DMSO, while DMSO was also loaded as control. The plates were kept for incubation at 37 °C for 24 h and then the plates were examined for the formation of zones of inhibition. Each inhibition zone was measured three times by caliper to get an average value. The test was performed three times for each bacterium and the average was taken. Ampicillin and Gentamicin were used as antibacterial standard drugs [68].

### 3.3. Molecular Docking

The docking studies of ligand to proteins active sites and estimating the binding affinities of docked compound with X-ray crystal Structure of *E. coli* 24 kDa Domain in complex with clorobiocin (PDB code: 1KZN) and the crystal structure of cytochrome P450 14𝛼-sterol demethylase (Cyp51) from *Mycobacterium tuberculosis* in complex with fluconazole (PDB 1EA1) were provided from Brookhaven protein data bank [69] and loaded to Molegro Virtual Docker (MVD2013.6.0.0 (win32)) program, fully functional free trial version with time limiting license [70] The non-bonded oxygen atoms of water, present in the crystal structure, was removed. MolDock score functions were used with a 0.3 Å grid resolution. The binding sites were defined to any residues with 10 Å distant from the cocrystallized clorobiocin and fluconazole in the complex crystal structure of the enzymes [69,70,71,72].

## 4. Conclusions

The synthesis and characterization of 2,6-bis(hydroxy(phenyl)methyl)cyclohexanone **1** is reported. The TGA analysis showed thermal stability of the studied compound up to 165 °C. The molecular structure of the studied compound has been optimized using the DFT/B3LYP method and 6-311G(d,p) basis set. The calculated bond distances and bond angles showed good agreement with our reported X-ray crystal structure. The molecular electrostatic potential picture of the studied compound has been calculated using the same level of theory. The MEP results showed that O1 and O3 are the most reactive H-acceptor sites while the H-atoms (H44 and H45) are the most reactive H-donor sites. The α_0_ and HOMO-LUMO energy gap (ΔE) values indicated that the studied molecule is better NLO material than urea. The calculated electronic spectra using the TD‒DFT method showed an intense transition band at 213.2 nm (f = 0.1792) due to H→L+1 (71%) and H−4→L+1(11%) excitations. The GIAO ^1^H- and ^13^C-NMR chemical shift values were correlated with the experimental data. The correlation coefficients (R^2^) for carbon and proton are 0.9957 and 0.9860, respectively. The Lewis structure NBO as well as the different ICT interactions in the studied molecule have been predicted using the NBO calculations. The polarity (3.9588D) of the studied compound is due to the high percentage polarization (81.60%–86.24%) at the O-atoms. The small LP(2)O2→BD*(1)O1-H44/BD*(1)O3-H45 indicate that the O···H intramolecular H-bonding interactions are weak. The anti-bacterial, anti-fungal and the molecular docking were investigated. Further studies of this molecule are currently under investigation in our laboratory.

## Supplementary Materials

Supplementary materials can be accessed at: http://www.mdpi.com/1420-3049/20/07/13240/s1.
