# Supplementary material for: Synthesis, Spectroscopic Investigations (X-ray, NMR and TD-DFT), Antimicrobial Activity and Molecular Docking of 2,6-Bis(hydroxy(phenyl)methyl)cyclohexanone
Source: Molecules. 2015 Jul 21;20(7):13240–63. doi: 10.3390/molecules200713240 (PMC6332055; doi:10.3390/molecules200713240)
Supplement: Supplementary file 1 [file molecules-20-13240-s001.pdf]

Supplementary Materials

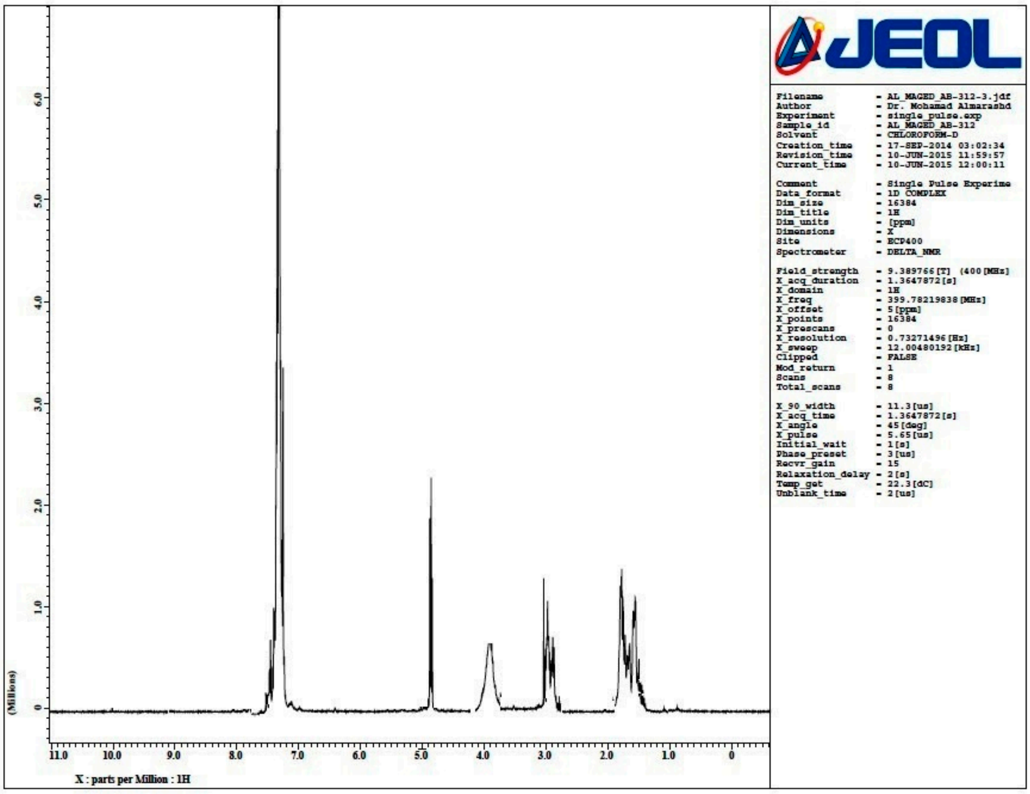

Figure S1.^{1}H-NMR of the compound **1**.

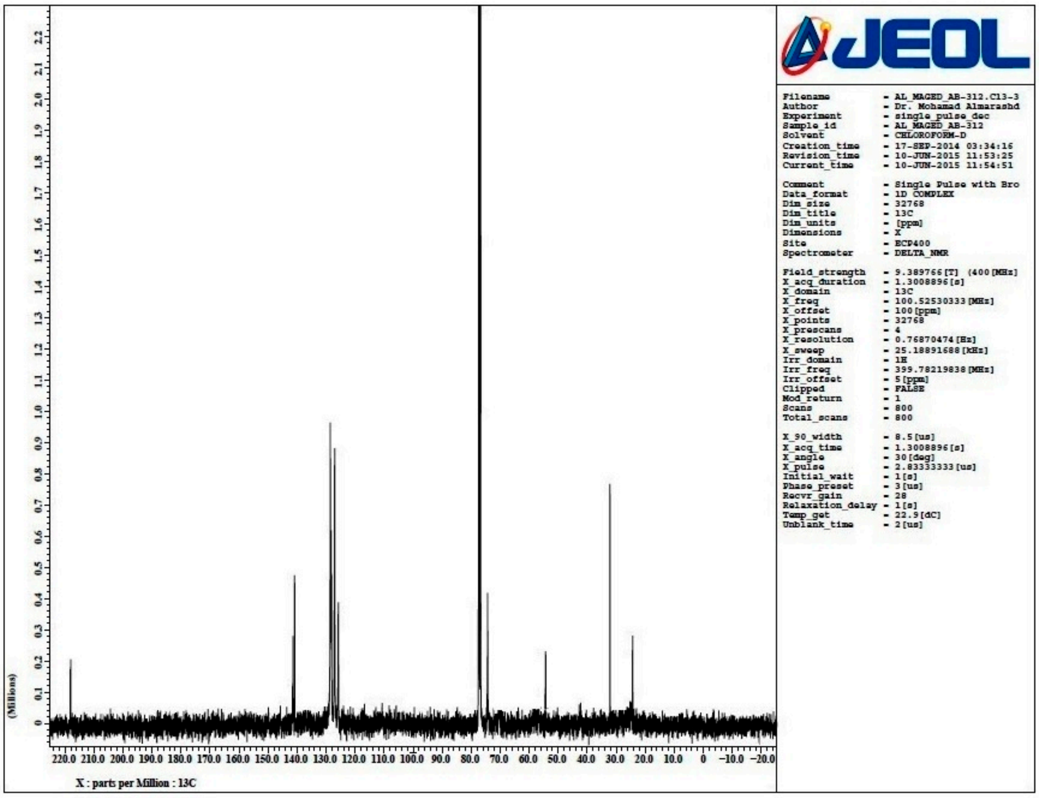

Figure S2.^{13}C-NMR of the compound **1**.

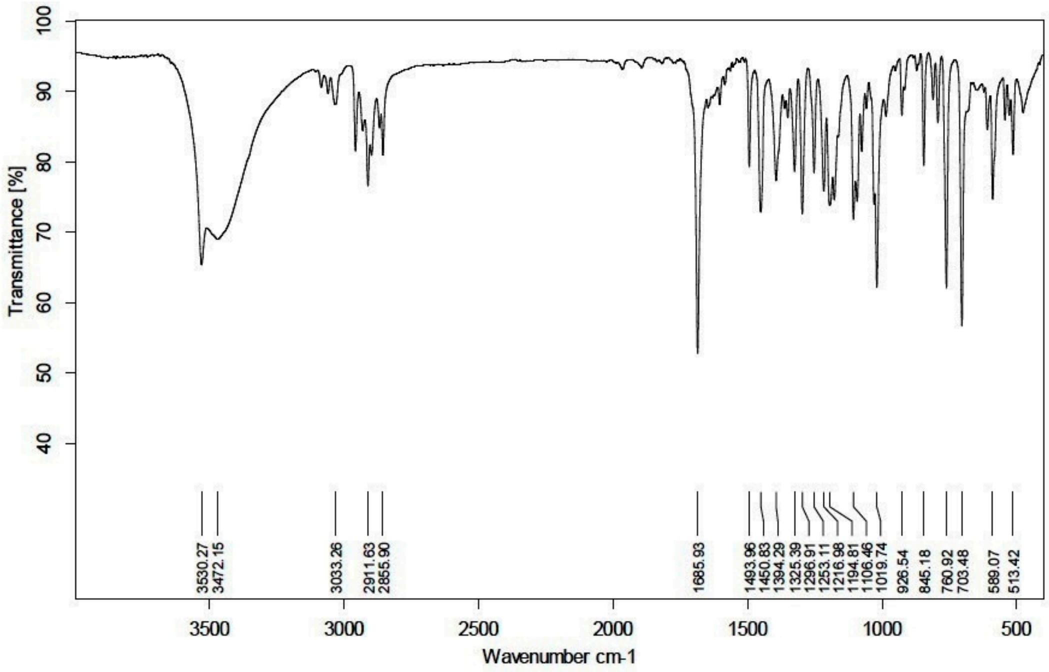

Figure S3.IR of the compound **1**.

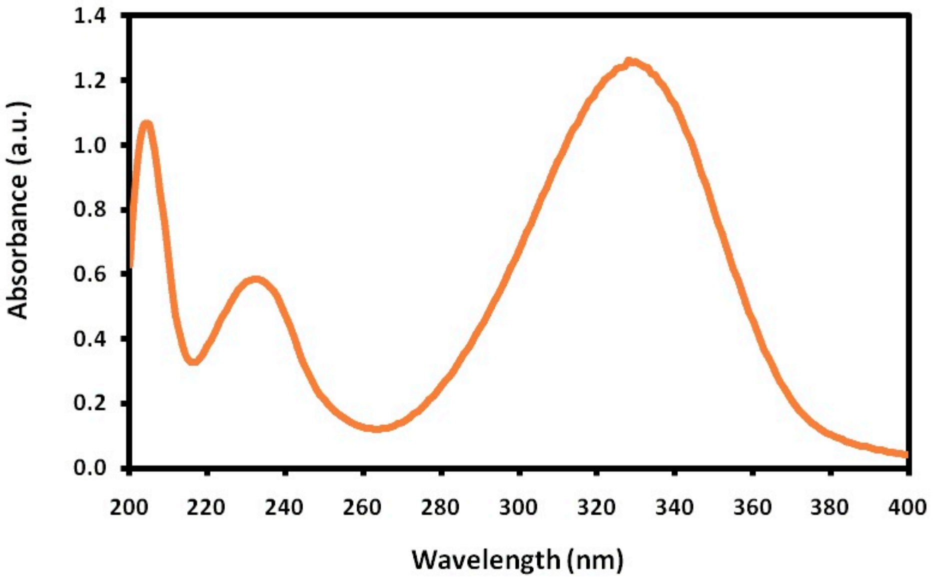

Figure S4. The experimental electronic spectra of **1**.

Table S1. Geometric parameters (Å, °) of **1**.

O1–C7	1.4257 (14)	C9–H9B	0.9700
O1–H1O1	0.896 (19)	C10–C11	1.5222 (15)
O2–C13	1.2240 (14)	C10–H10A	0.9700
O3–C14	1.4334 (14)	C10–H10B	0.9700
O3–H1O3	0.90 (2)	C11–C12	1.5392 (16)
C1–C2	1.3930 (16)	C11–H11A	0.9700
C1–C6	1.3944 (16)	C11–H11B	0.9700
C1–H1A	0.9300	C12–C13	1.5215 (14)
C2–C3	1.389 (2)	C12–C14	1.5379 (14)
C2–H2A	0.9300	C12–H12A	0.9800
C3–C4	1.386 (2)	C14–C15	1.5128 (15)
C3–H3A	0.9300	C14–H14A	0.9800
C4–C5	1.3957 (17)	C15–C20	1.3913 (16)
C4–H4A	0.9300	C15–C16	1.3941 (16)
C5–C6	1.3921 (17)	C16–C17	1.3895 (16)
C5–H5A	0.9300	C16–H16A	0.9300
C6–C7	1.5156 (15)	C17–C18	1.3923 (19)
C7–C8	1.5396 (15)	C17–H17A	0.9300
C7–H7A	0.9800	C18–C19	1.3854 (18)
C8–C13	1.5219 (15)	C18–H18A	0.9300
C8–C9	1.5412 (16)	C19–C20	1.3950 (16)
C8–H8A	0.9800	C19–H19A	0.9300
C9–C10	1.5240 (16)	C20–H20A	0.9300
C9–H9A	0.9700		
C7–O1–H1O1	107.6 (13)	C9–C10–H10B	109.3
C14–O3–H1O3	105.0 (14)	H10A–C10–H10B	107.9
C2–C1–C6	120.56 (12)	C10–C11–C12	111.89 (9)
C2–C1–H1A	119.7	C10–C11–H11A	109.2
C6–C1–H1A	119.7	C12–C11–H11A	109.2
C3–C2–C1	120.23 (12)	C10–C11–H11B	109.2
C3–C2–H2A	119.9	C12–C11–H11B	109.2
C1–C2–H2A	119.9	H11A–C11–H11B	107.9
C4–C3–C2	119.52 (11)	C13–C12–C14	112.27 (9)
C4–C3–H3A	120.2	C13–C12–C11	109.07 (9)
C2–C3–H3A	120.2	C14–C12–C11	111.71 (9)
C3–C4–C5	120.35 (12)	C13–C12–H12A	107.9
C3–C4–H4A	119.8	C14–C12–H12A	107.9
C5–C4–H4A	119.8	C11–C12–H12A	107.9
C6–C5–C4	120.42 (12)	O2–C13–C12	121.73 (9)
C6–C5–H5A	119.8	O2–C13–C8	121.94 (10)
C4–C5–H5A	119.8	C12–C13–C8	116.29 (9)
C5–C6–C1	118.92 (10)	O3–C14–C15	107.91 (9)
C5–C6–C7	120.88 (10)	O3–C14–C12	111.31 (9)
C1–C6–C7	120.17 (10)	C15–C14–C12	110.83 (9)
O1–C7–C6	107.42 (9)	O3–C14–H14A	108.9
O1–C7–C8	110.81 (9)	C15–C14–H14A	108.9
C6–C7–C8	111.99 (9)	C12–C14–H14A	108.9
O1–C7–H7A	108.9	C20–C15–C16	119.16 (10)

Table S1. *Cont.*

C6–C7–H7A	108.9	C20–C15–C14	120.74 (10)
C8–C7–H7A	108.9	C16–C15–C14	120.01 (10)
C13–C8–C7	112.02 (9)	C17–C16–C15	120.44 (11)
C13–C8–C9	108.48 (9)	C17–C16–H16A	119.8
C7–C8–C9	113.39 (9)	C15–C16–H16A	119.8
C13–C8–H8A	107.6	C16–C17–C18	120.12 (11)
C7–C8–H8A	107.6	C16–C17–H17A	119.9
C9–C8–H8A	107.6	C18–C17–H17A	119.9
C10–C9–C8	111.70 (9)	C19–C18–C17	119.74 (11)
C10–C9–H9A	109.3	C19–C18–H18A	120.1
C8–C9–H9A	109.3	C17–C18–H18A	120.1
C10–C9–H9B	109.3	C18–C19–C20	120.12 (11)
C8–C9–H9B	109.3	C18–C19–H19A	119.9
H9A–C9–H9B	107.9	C20–C19–H19A	119.9
C11–C10–C9	111.66 (9)	C15–C20–C19	120.40 (11)
C11–C10–H10A	109.3	C15–C20–H20A	119.8
C9–C10–H10A	109.3	C19–C20–H20A	119.8
C11–C10–H10B	109.3		

Table S2. Torsion angles of **1**.

C6–C1–C2–C3	0.52 (18)	C11–C12–C13–O2	−124.14 (11)
C1–C2–C3–C4	0.29 (19)	C14–C12–C13–C8	177.99 (9)
C2–C3–C4–C5	−0.67 (19)	C11–C12–C13–C8	53.62 (12)
C3–C4–C5–C6	0.25 (18)	C7–C8–C13–O2	−2.35 (15)
C4–C5–C6–C1	0.56 (17)	C9–C8–C13–O2	123.58 (12)
C4–C5–C6–C7	−177.56 (11)	C7–C8–C13–C12	179.90 (9)
C2–C1–C6–C5	−0.94 (17)	C9–C8–C13–C12	−54.18 (12)
C2–C1–C6–C7	177.19 (11)	C13–C12–C14–O3	57.22 (12)
C5–C6–C7–O1	140.61 (11)	C11–C12–C14–O3	−179.89 (9)
C1–C6–C7–O1	−37.48 (13)	C13–C12–C14–C15	177.33 (9)
C5–C6–C7–C8	−97.50 (12)	C11–C12–C14–C15	−59.78 (12)
C1–C6–C7–C8	84.41 (13)	O3–C14–C15–C20	−136.66 (10)
O1–C7–C8–C13	−61.83 (12)	C12–C14–C15–C20	101.22 (12)
C6–C7–C8–C13	178.24 (9)	O3–C14–C15–C16	46.76 (13)
O1–C7–C8–C9	174.97 (9)	C12–C14–C15–C16	−75.36 (13)
C6–C7–C8–C9	55.05 (13)	C20–C15–C16–C17	−1.46 (16)
C13–C8–C9–C10	53.92 (12)	C14–C15–C16–C17	175.17 (10)
C7–C8–C9–C10	179.04 (9)	C15–C16–C17–C18	0.37 (18)
C8–C9–C10–C11	−57.12 (13)	C16–C17–C18–C19	0.88 (18)
C9–C10–C11–C12	56.31 (13)	C17–C18–C19–C20	−1.02 (18)
C10–C11–C12–C13	−52.59 (12)	C16–C15–C20–C19	1.32 (17)
C10–C11–C12–C14	−177.29 (9)	C14–C15–C20–C19	−175.29 (10)
C14–C12–C13–O2	0.22 (15)	C18–C19–C20–C15	−0.09 (18)

All e.s.d.'s (except the e.s.d. in the dihedral angle between two l.s. planes) are estimated using the full covariance matrix. The cell e.s.d.'s are taken into account individually in the estimation of e.s.d.'s in distances, angles and torsion angles; correlations between e.s.d.'s in cell parameters are only used when they are defined by crystal symmetry. An approximate (isotropic) treatment of cell e.s.d.'s is used for estimating e.s.d.'s involving l.s. planes.

Table S3. The calculated electronic transition bands using TD-DFT method.

λ_{max} (nm)	f	Major Contributions
294.6	0.0010	H-6→L (15%), H-4→L (41%), H→L (38%)
256.4	0.0005	H-6→L (28%), H-4→L (12%), H→L (55%)
255.9	0.0008	H-5→L (15%), H-1→L (81%)
244.3	0.0002	H-3→L (80%), H-1→L (10%)
243.8	0.0039	H-2→L (88%)
238.6	0.0004	H-6→L (31%), H-5→L (29%), H-4→L (22%)
238.6	0.0003	H-6→L (20%), H-5→L (45%), H-4→L (14%)
230.7	0.0000	H-3→L+4 (15%), H-2→L+1 (26%), H-1→L+2 (17%), H→L+3 (29%)
230.7	0.0003	H-3→L+1 (25%), H-2→L+4 (15%), H-1→L+3 (22%), H→L+2 (23%)
**213.2**	**0.1792**	**H-4→L+1 (11%), H→L+1 (71%)**
209.1	0.0092	H-1→L+1 (13%), H→L+2 (10%), H→L+4 (38%)
203.6	0.0029	H-4→L+2 (16%), H-1→L+1 (24%), H→L+2 (37%)
203.5	0.0008	H-4→L+3 (22%), H-1→L+2 (17%), H→L+3 (45%)
203.0	0.0039	H-1→L+1 (36%), H→L+4 (30%)
201.3	0.0085	H-4→L+1 (22%), H-3→L+2 (12%), H-2→L+3 (14%), H-1→L+4 (34%)
199.8	0.0001	H-5→L+4 (11%), H-4→L+1 (26%), H-1→L+4 (22%), H→L+1 (14%)
199.5	0.0009	H-5→L+1 (38%), H-4→L+4 (12%)
197.5	0.0029	H-5→L+3 (10%), H-4→L+3 (12%), H-1→L+2 (15%), H-1→L+3 (17%)
197.5	0.0030	H-5→L+3 (12%), H-4→L+3 (13%), H-1→L+2 (13%), H-1→L+3 (19%)
195.7	0.0011	H-3→L+2 (13%), H-3→L+4 (32%), H-2→L+1 (47%)

Table S4. The calculated chemical shifts δ (ppm) of the studied compound using GIAO method.

Atom	δ_{calc} (ppm)	δ_{exp}.(ppm)	Atom	δ_{calc} (ppm)	δ_{exp}.(ppm)
C4	132.40	128.28	H5	7.88	7.35
C6	134.49	128.28	H7	7.57	7.35
C8	133.18	127.81	H 9	7.43	7.25
C10	132.30	128.28	H11	7.37	7.25
C12	133.07	128.28	H13	7.13	7.25
C14	149.23	141.01	H16	4.81	4.83
C15	81.12	75.06	H18	2.16	3.01
C17	66.49	54.51	H20	1.53	1.70
C19	36.41	31.36	H21	1.22	1.20
C22	29.28	20.75	H23	1.31	1.20
C25	36.41	31.36	H24	1.54	1.70
C28	66.48	54.51	H26	1.21	1.20
C30	233.38	217.06	H27	1.52	1.70
C31	81.14	75.06	H29	2.17	3.01
C33	149.23	141.01	H 32	4.81	4.83
C34	132.40	128.28	H35	7.88	7.35
C36	134.50	128.28	H37	7.57	7.35
C38	133.19	127.81	H39	7.44	7.25
C40	132.30	128.28	H41	7.37	7.25
C42	133.08	128.28	H43	7.14	7.25
			H44	3.41	4.01
			H45	3.42	4.01

Table S5. Natural bond orbital analyses of the studied compound.

BOND (A–B)	ED	ED_A (%)	ED_B (%)	$(NBO)_A$	$(NBO)_B$	S (%)		P (%)	
						A	B	A	B
BD (1) O1-C15	1.9891	66.59	33.41	0.8160	0.5780	30.15	20.40	69.79	79.37
BD (1) O1-H44	1.9869	74.37	25.63	0.8624	0.5062	21.32	99.82	78.59	0.18
BD (1) O2-C30	1.9943	67.29	32.71	0.8203	0.5720	44.21	28.77	55.69	71.04
BD (2) O2-C30	1.9832	69.76	30.24	0.8352	0.5499	0.69	0.39	99.20	99.14
BD (1) O3-C31	1.9891	66.59	33.41	0.8160	0.5780	30.15	20.40	69.79	79.37
BD (1) O3-H45	1.9869	74.37	25.63	0.8624	0.5062	21.32	99.82	78.59	0.18
BD (1) C4-H5	1.9781	60.98	39.02	0.7809	0.6247	27.95	99.95	72.00	0.05
BD (1) C4-C6	1.9770	50.24	49.76	0.7088	0.7054	35.90	36.26	64.06	63.70
BD (2) C4-C6	1.6611	49.61	50.39	0.7044	0.7098	0.01	0.00	99.95	99.96
BD (1) C4-C14	1.9713	49.42	50.58	0.7030	0.7112	36.12	34.30	63.84	65.65
BD (1) C6-H7	1.9796	60.17	39.83	0.7757	0.6311	27.68	99.95	72.27	0.05
BD (1) C6-C8	1.9790	49.99	50.01	0.7071	0.7071	36.02	36.04	63.94	63.92
BD (1) C8-H9	1.9797	60.16	39.84	0.7756	0.6312	27.89	99.95	72.06	0.05
BD (1) C8-C10	1.9787	49.91	50.09	0.7065	0.7077	36.04	36.12	63.92	63.84
BD (2) C8-C10	1.6683	50.11	49.89	0.7079	0.7064	0.00	0.00	99.96	99.96
BD (1) C10-H11	1.9795	60.16	39.84	0.7756	0.6312	27.70	99.95	72.24	0.05
BD (1) C10-C12	1.9769	49.67	50.33	0.7047	0.7095	36.14	35.93	63.82	64.02
BD (1) C12-H13	1.9793	60.15	39.85	0.7756	0.6312	27.37	99.95	72.58	0.05
BD (1) C12-C14	1.9720	49.52	50.48	0.7037	0.7105	36.65	34.78	63.32	65.17
BD (2) C12-C14	1.6626	50.19	49.81	0.7084	0.7058	0.01	0.04	99.95	99.92
BD (1) C14-C15	1.9684	50.20	49.80	0.7085	0.7057	30.85	29.61	69.12	70.35
BD (1) C15-H16	1.9763	59.32	40.68	0.7702	0.6378	22.15	99.97	77.77	0.03
BD (1) C15-C17	1.9674	48.22	51.78	0.6944	0.7196	27.86	27.63	72.09	72.34
BD (1) C17-H18	1.9468	61.25	38.75	0.7826	0.6225	19.85	99.98	80.07	0.02
BD (1) C17-C19	1.9593	51.39	48.61	0.7169	0.6972	26.93	27.59	73.03	72.38
BD (1) C17-C30	1.9741	51.44	48.56	0.7172	0.6968	25.61	35.38	74.32	64.59
BD (1) C19-H20	1.9760	61.04	38.96	0.7813	0.6241	22.41	99.97	77.52	0.03
BD (1) C19-H21	1.9790	59.72	40.28	0.7728	0.6347	21.40	99.97	78.52	0.03
BD (1) C19-C22	1.9798	50.42	49.58	0.7101	0.7041	28.55	28.25	71.41	71.71
BD (1) C22-H23	1.9781	59.71	40.29	0.7727	0.6347	21.58	99.96	78.35	0.04
BD (1) C22-H24	1.9756	60.28	39.72	0.7764	0.6302	21.86	99.96	78.07	0.04
BD (1) C22-C25	1.9798	49.58	50.42	0.7041	0.7101	28.25	28.55	71.71	71.41
BD (1) C25-H26	1.9790	59.72	40.28	0.7728	0.6347	21.40	99.97	78.52	0.03
BD (1) C25-H27	1.9760	61.04	38.96	0.7813	0.6242	22.41	99.97	77.52	0.03
BD (1) C25-C28	1.9593	48.61	51.39	0.6972	0.7169	27.59	26.93	72.38	73.03
BD (1) C28-H29	1.9468	61.25	38.75	0.7826	0.6225	19.85	99.98	80.07	0.02
BD (1) C28-C30	1.9741	51.45	48.55	0.7173	0.6968	25.61	35.38	74.32	64.59
BD (1) C28-C31	1.9674	51.78	48.22	0.7196	0.6944	27.63	27.86	72.34	72.10
BD (1) C31-H32	1.9763	59.32	40.68	0.7702	0.6378	22.15	99.97	77.77	0.03
BD (1) C31-C33	1.9684	49.80	50.20	0.7057	0.7085	29.60	30.85	70.35	69.12
BD (1) C33-C34	1.9713	50.58	49.42	0.7112	0.7030	34.30	36.13	65.66	63.84
BD (1) C33-C42	1.9720	50.48	49.52	0.7105	0.7037	34.79	36.65	65.17	63.32
BD (2) C33-C42	1.6627	49.82	50.18	0.7058	0.7084	0.04	0.01	99.92	99.95
BD (1) C34-H35	1.9781	60.97	39.03	0.7809	0.6247	27.95	99.95	72.00	0.05
BD(1) C34-C36	1.9770	50.24	49.76	0.7088	0.7054	35.90	36.26	64.06	63.70

Table S5. *Cont.*

BOND(A–B)	ED	ED_A (%)	ED_B (%)	$(NBO)_A$	$(NBO)_B$	S (%)		P (%)	
						A	B	A	B
BD(2) C34-C36	1.6612	49.61	50.39	0.7044	0.7098	0.01	0.00	99.95	99.96
BD(1) C36-H37	1.9796	60.17	39.83	0.7757	0.6311	27.68	99.95	72.27	0.05
BD(1) C36-C38	1.9790	49.99	50.01	0.7071	0.7071	36.02	36.04	63.94	63.92
BD(1) C38-H39	1.9797	60.16	39.84	0.7756	0.6312	27.89	99.95	72.06	0.05
BD(1) C38-C40	1.9787	49.91	50.09	0.7065	0.7077	36.04	36.12	63.92	63.84
BD(2) C38-C40	1.6683	50.11	49.89	0.7079	0.7064	0.00	0.00	99.96	99.96
BD(1) C40-H41	1.9795	60.16	39.84	0.7756	0.6312	27.71	99.95	72.24	0.05
BD(1) C40-C42	1.9769	49.67	50.33	0.7048	0.7095	36.13	35.93	63.82	64.02
BD(1) C42-H43	1.9793	60.16	39.84	0.7756	0.6312	27.37	99.95	72.58	0.05
LP(1)O1	1.9795					48.51		51.46	
LP(2)O1	1.9524					0.01		99.95	
LP(1)O2	1.9692					55.08		44.90	
LP(2)O2	1.8917					0.00		99.95	
LP(1)O3	1.9795					48.51		51.46	
LP(2)O3	1.9524					0.01		99.95	
